# A Survey of LoRaWAN for IoT: From Technology to Application

**DOI:** 10.3390/s18113995

**Published:** 2018-11-16

**Authors:** Jetmir Haxhibeqiri, Eli De Poorter, Ingrid Moerman, Jeroen Hoebeke

**Affiliations:** IDLab, Department of Information Technology at Ghent University—IMEC, 9052 Ghent, Belgium; eli.depoorter@ugent.be (E.D.P.); ingrid.moerman@ugent.be (I.M.); jeroen.hoebeke@ugent.be (J.H.)

**Keywords:** LoRaWAN, LoRa, IoT, LPWANs, IoT applications

## Abstract

LoRaWAN is one of the low power wide area network (LPWAN) technologies that have received significant attention by the research community in the recent years. It offers low-power, low-data rate communication over a wide range of covered area. In the past years, the number of publications regarding LoRa and LoRaWAN has grown tremendously. This paper provides an overview of research work that has been published from 2015 to September 2018 and that is accessible via Google Scholar and IEEE Explore databases. First, a detailed description of the technology is given, including existing security and reliability mechanisms. This literature overview is structured by categorizing papers according to the following topics: (i) physical layer aspects; (ii) network layer aspects; (iii) possible improvements; and (iv) extensions to the standard. Finally, a strengths, weaknesses, opportunities and threats (SWOT) analysis is presented along with the challenges that LoRa and LoRaWAN still face.

## 1. Introduction

Internet of Things (IoT) applications need more and more technologies that can offer low-power operation, low-cost and low-complexity end devices that will be able to communicate wirelessly over large distances. As in most cases the IoT end devices are battery-powered sensor nodes, the power usage profile should be carefully designed in order to extend the battery lifetime. Communication range need to go from several hundreds of meters up to several kilometers, as end devices are distributed over large area of operation. Considering all the aforementioned characteristics, this can be only be realized by using low power wide area network (LPWAN) technologies.

Several LPWAN technologies are already present in the market: SigFox, NB-IoT or LoRaWAN. SigFox plans to offer global coverage (currently, September 2018, 45 covered countries and regions) by means of a single operator network, with roll-outs in different countries performed by a number of member companies. NB-IoT is being offered by telecommunication companies as an IoT communication alternative to sub-GHz LPWAN technologies. As NB-IoT operates in licensed spectrum, it offers higher traffic reliability compared to other sub-GHz technologies. Unlike SigFox and NB-IoT, LoRaWAN offers the possibility for private network deployments and easy integration with a number of world-wide network platforms (e.g., The Things Network). Due to this and its open access specifications, LoRaWAN drew the research community’s attention since its first appearance in the market.

The research community started to study LoRa and LoRaWAN performance and different technology characteristics as early as in 2015. Since then, numerous research papers have been published in different peer reviewed journals and presented in scientific conferences all over the world. On September 2018, there were around 2000 publications listed by the Google Scholar database that contained the keyword “LoRaWAN” in their text. At the same time, in the IEEE Explore database, there were 162 papers in total that contained “LoRaWAN” as keyword in their title. Of these, 18 were published in IEEE journals and magazines while the rest were published in IEEE conference proceedings.

As the number of publications is large, this paper presents a structured literature review of LoRa and LoRaWAN published papers. The literature search was done in Google Scholar and IEEE Explore databases and it includes the most relevant studies from 2015 to September 2018. The literature selection was based on the integrity of the journal and conference where the research work was published/presented.

This paper is organized as follows. First, we give a detailed introduction to LoRa and LoRaWAN technologies in Section 2. Section 2.2.1 and Section 2.2.2 detail the most relevant algorithms in LoRaWAN: the adaptive data rate mechanism and the end node join procedure with security context. Section 3 offers a literature review of applications and deployments that are based on LoRa and LoRaWAN. Section 4 details the work that is done in designing network level simulators for LoRaWAN as well as deployed LoRaWAN testbeds. Section 5 and Section 6 overview the research done regarding the performance evaluation of the LoRa physical layer and LoRaWAN, respectively. The physical layer performance section includes studies related to coverage tests and impact of interference on LoRa. The network layer performance section reviews studies related to the design of LoRaWAN MAC layer models, power usage studies as well as security related studies. Section 7 offers a review of studies related to a number of improvements introduced to LoRaWAN for better performance, including here new ADR mechanisms, transmission synchronization and scheduling possibilities. In Section 8, different feature extensions to LoRa and LoRaWAN are detailed. It includes studies related to new MAC design on top of LoRa, multi-hop LoRa communications and IP communication over LoRaWAN. Section 9 details open challenges of LoRa and LoRaWAN, including a SWOT analysis of the technology. Section 10 concludes the paper.

## 2. Introduction to LoRa and LoRaWAN

This section gives a detailed overview of the technology, covering the LoRa physical layer as well as the LoRaWAN MAC layer open standard.

### 2.1. LoRa

The LoRa physical layer has been patented by Semtech in 2014 [1]. Its modulation is based on [2] and produces a chirp signal where all chirps will have practically the same time duration. A chirp is characterized by a time profile of the instantaneous frequency that changes over the time interval *T* from a frequency $f_{0}$ to $f_{1}$. In LoRa, two different types of chirps have been defined [1]: the base chirp whose frequency time profile starts with the minimal frequency $f_{min}=-\frac{BW}{2}$ and ends with the maximal frequency $f_{max}=+\frac{BW}{2}$, BW being the spreading bandwidth of the signal, and modulated chirps that are cyclically time shifted base chirps. The chirp that starts with frequency $f_{1}=+\frac{BW}{2}$ and ends with $f_{2}=-\frac{BW}{2}$ is referred to as a down-chirp, practically being the complex conjugate of the base chirp. For different digital inputs, a modulator will produce different chirps that will have certain time shift compared to base chirp.

The time shift of each chirp at the receiver side can be determined after the alignment of the time reference between receiver and transmitter by means of preamble detection. The time shift of each chirp is determined by multiplying the chirp itself with a down-chirp and finding the Fast Fourier Transform (FFT) of the multiplication. The maximum of the FFT output will indicate the time shift of the transmitted chirp that consequently will determine the transmitted digital symbol. If *N* is the chirp length of a symbol, then there can be *N* possible different cyclic shifts of the base chirp, and the value of the cyclic shift can be coded using $log_{2}N$ bits that gives the spreading factor (SF) of the communication.

To further improve the robustness against noise and burst interference, LoRa uses diagonal interleaving as well as forward error correction (FEC) codes with code rates from 4/5 to 4/8. The data rate and symbol rate depends on the SF and the bandwidth used. The symbol rate is given by the formula:

(1) $$R_{s}=SF\cdot \frac{BW}{2^{SF}}$$

where SF is the spreading factor and BW is the bandwidth in Hz. From Equation (1), it is seen that the symbol time is increased by increasing the SF, decreasing the symbol rate. The data rate is given by the formula:

(2) $$R_{b}=SF\cdot \frac{\frac{4}{4+CR}}{\frac{2^{SF}}{BW}}\cdot 1000$$

where SF is the spreading factor, CR is the code rate and BW is the bandwidth in KHz. The data rate in this formula is expressed in bps. From Equation (2), it can be noticed that by increasing the SF less bits per symbol will be encoded, decreasing the data rate.

**Figure 1:**
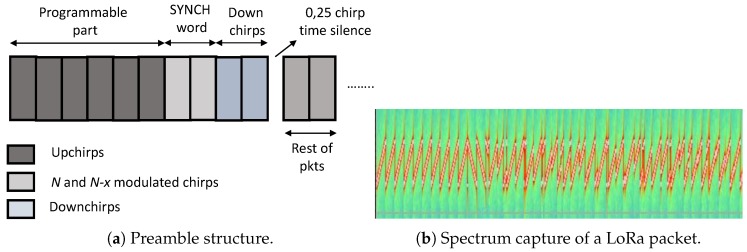
LoRa preamble.

The packet structure at the physical layer includes a preamble, an optional header and the data payload. The preamble is used to synchronize the receiver with the transmitter and can have a length from 10 up to 65,536 symbols in total. The fixed part of the preamble consists of four symbols, and the rest is programmable with a minimal length of six symbols and a maximal length of 65,532 [3]. According to Seller and Sornin [1], the preamble starts with a sequence of constant upchirp symbols that is programmable and helps to detect the start of the frame. The programmable part is followed by two chirp symbols encoding the sync word that is used for frame synchronization. Usually, the two chirps will modulate opposite values, e.g., *x* and N−x. When the receiver detects three consecutive chirps, with the first one unmodulated (upchirp), the second one modulated with the first value *x* and the third one modulated with the opposite value N−x), a new frame is detected. The sync word can also be used to distinguish between devices from different networks by using different values for each network. Next to this, the preamble ends with two downchirp symbols that are used for frequency synchronization. After the last two symbols, a 0.25 symbol time represents a silence time used to let the receiver align the time [1]. Optionally, the end of preamble can include another two unmodulated base chirps that will be used for fine time and frequency synchronization [1]. The structure of the preamble is shown in Figure 1a, while Figure 1b shows the spectrum capture of a LoRa packet where upchirps at the beginning of preamble are easily noticeable. Note that transmitter and receiver should know the SF in advance in order to detect the preamble, as preamble size scales with SF and there is no single preamble for all SFs [1].

**Figure 2:**
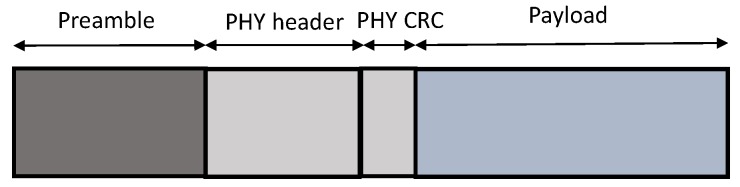
LoRa frame structure.

Optionally, the preamble is followed by a physical header. In such a case, the header contains the payload length in bytes, the FEC code rate of the payload and the header CRC. The header is always protected with FEC with highest code rate of 4/8. If these three parameters are known in advance, the header can be removed completely. This decreases the time on air of the packet. In this case, the implicit header mechanism is applied, where the header parameters are fixed beforehand at both ends, the receiver and the transmitter side. The payload contains either LoRaWAN MAC layer control packets or data packets. Optionally, the payload can be followed by a payload CRC. The frame structure is shown in Figure 2.

As previously said, the LoRa modulation mechanism has been patented and its implementation is never made public. The first mathematical description on how LoRa modulation works is presented in [4]. The authors described the LoRa modulation as Frequency Shift Chirp Modulation (FSCM) and give the mathematical model for it.

The LoRa physical layer operates in the 433-, 868- or 915-MHz frequency bands. In Europe, only the 868- and 433-MHz bands can be used. In the 868-MHz band, there are three 125-kHz channels that are mandatory to be implemented in every end device. There are another five 125-kHz channels in the 867-MHz sub-band that can be optionally used for LoRa communication [5]. In Europe due to transmission regulations [6], each transmission in any of the 868 MHz and 867 MHz sub-bands should comply with a 1% radio duty cycle or implement a listen-before-talk or adaptive frequency agility mechanism. When the duty cycle regulation is followed, it means that, if the radio transmitted for 1 s, it cannot transmit for the next 99 s.

### 2.2. LoRaWAN MAC Layer

The LoRaWAN medium access control (MAC) protocol is an open source protocol standardized by the LoRa Alliance [7] that runs on top of LoRa [1] physical layer. The LoRaWAN MAC layer provides the medium access control mechanism that enables communication between multiple devices and network gateway(s).

**Figure 3:**
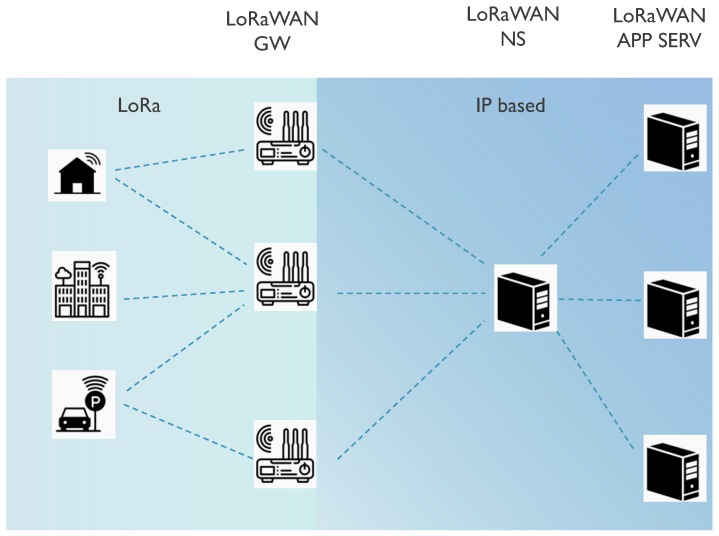
LoRaWAN network topology.

The LoRaWAN network architecture has a star topology, where the end devices can only communicate with LoRaWAN gateways and not directly with each other. Multiple gateways are connected to a central network server. The LoRaWAN gateways are only responsible for forwarding raw data packets from end nodes towards the network server encapsulating them in UDP/IP packets. The network server is responsible for sending downlink packets and MAC commands towards end devices, if needed. Further, the communication terminates at the application servers that can be owned by third parties. Multiple application layers can be connected to a single network server. The resulting LoRaWAN network architecture is shown in Figure 3.

The LoraWAN standard defines three classes of end devices, Classes A–C. Features of Class A devices are basic sets of options that every end device needs to implement in order to join a LoRaWAN network. To enable bidirectional communication, each uplink transmission of a Class A device is followed by two short downlink receive windows during which the end device will listen for possible downlink traffic. Consequently, the downlink communication is triggered by the end device, meaning that each downlink frame needs to wait for an uplink communication. The first and second downlink receive windows start 1 and 2 s, respectively, after the end of the uplink transmission. If the downlink transmission happens during the first window then the same channel that is used for uplink is used for downlink too, while SF is determined based on the *RX1DROffset* parameter [8]. In case the second receive window is used for the downlink transmission, then a fixed SF and channel are used. This is typically the 125 kHz channel centered at 869.525 MHz using SF12, which has a 10% duty cycle and high transmit power of 24 dBm. It is the responsibility of the network server to schedule the downlink traffic at the exact time and to perform the timing control. Class A end devices consume the least power since most of the time they are asleep.

To increase the downlink possibilities, Class B end devices will open additional receive windows at scheduled times. Gateways will transmit beacons in downlink for Class B end devices to get synchronized and for the network server to know when a certain end device will listen for downlink traffic. Class B devices consume higher power compared to Class A devices as they need to open more receive windows, even if those windows might not be used for downlink traffic at all. Class C devices will open continuous receive windows, practically being all the time available for downlink traffic except the time when they are transmitting [9].

LoRaWAN protocol specifies a number of mechanisms that ensures reliable and secure communication. In the following subsections, we describe the adaptive data rate (ADR) mechanism as well as network joining procedures for end nodes.

#### 2.2.1. Adaptive and Data Rate Mechanism

An Adaptive Data Rate (ADR) mechanism is built into LoRaWAN for dynamically managing an end node’s link parameters in order to increase the packet delivery ratio. The ADR mechanism manages the data rate and transmit power of end devices. If the device wants to allow network server to manage its transmit parameters, it will set the uplink ADR bit in its uplink communication packets. Otherwise, the end device has the possibility to manage its transmit parameters itself by making use of the ADR mechanism that resides at the end device side. Thus, both parts of the ADR mechanism run asynchronously at the network server and at the end node.

**Figure 4:**
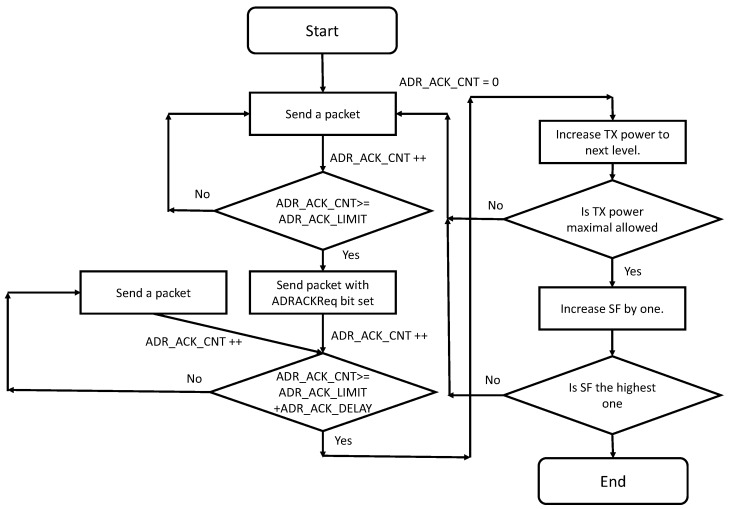
ADR mechanism algorithm implemented in the end node.

According to the latest release of the LoRaWAN standard (v1.1), the ADR algorithm at the end node is simple. It includes two parameters ADR_ACK_LIMIT and ADR_ACK_DELAY that are specified according to lor [10] and that have been set to be 64 and 32, respectively. The end device will increment ADR_ACK_CNT for every uplink packet that is sent. Once the ADR_ACK_CNT will become higher than ADR_ACK_LIMIT, the end device will set the ADRACKReq bit and will wait for an acknowledgment from the network for the next ADR_ACK_DELAY uplink packets. If there is no acknowledgment before ADR_ACK_DELAY uplink packets, the end device will decrease the data rate trying to regain connectivity to the network [9]. According to the newest standard, the end device first tries to gain connectivity by increasing its transmit power. If this is not sufficient, it will continue to decrease the data rate as part of the next step [11]. In Figure 4, the ADR mechanism implemented in the end node is shown.

The ADR mechanism in the network server determines the transmission parameters (SF and transmit power) of the end node based on the estimation of the link budget in the uplink and the threshold SNR for decoding the packet correctly at the current data rate.

#### 2.2.2. Network Joining Mechanism and Security Context

The LoRaWAN standard specifies how an end node can join the network. Each end device that joins the network needs to be personalized and activated. End devices can be activated over the air (OTAA) or by personalization (ABP).

Several keys and identifiers are needed for the joining procedure, as well as for all communication during one session. All the required keys and identifiers are shown in Table 1. The session keys are valid only for a single communication session between an end device and the network server/application server. It is desirable that session keys are updated by a rejoining procedure before the saturation of packet counters [11] to avoid any possible replaying attack.

**Figure 5:**
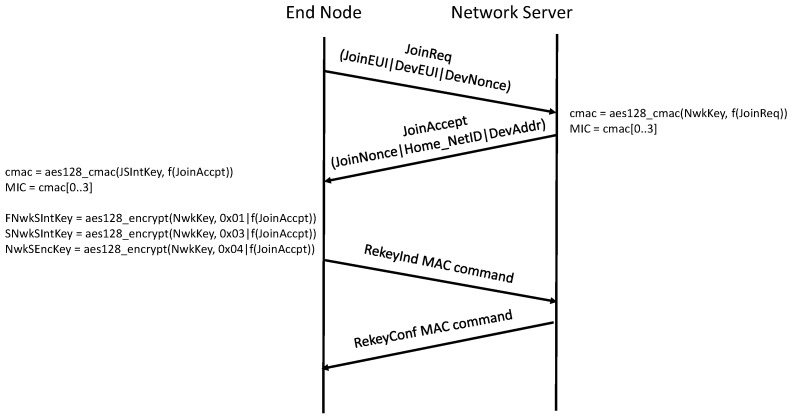
Over the air activation procedure according to lor [11].

When using the OTAA procedure, an end device is personalized with keys and identifiers that are saved before the procedure starts as shown in Table 1. The end node can send join or re-join requests, whereas the network server will reply with join-accept messages. A join-request message will include the DevEUI, JoinEUI IDs as well as 2 bytes of a DevNonce, which is a counter-based nonce. The join-request is not encrypted, but the message integrity check (MIC) is calculated using NwkKey. The network server will respond with a join-accept message if the device is permitted to join the network. The join-accept message will contain DevAddr and 3 bytes of JoinNonce among other communication parameters. The JoinNonce is an incremental number that will not repeat. The join-accept message is encrypted using NwkKey (if it is a reply to join-request message) or JSEncKey (if it is a reply to rejoin-request message). Its MIC is verified using JSIntKey. The join-accept message is accepted if the MIC is verified and the JoinNonce is greater than the last stored one. Finally, the FNwkSIntKey, SNwkSIntKey and NwkSEncKey are derived using NwkKey, while AppSKey is derived using AppKey. At the end, the end device will send a MAC command to the network server to confirm switching to the new security context. The new security context will be used once the network server acknowledges this MAC command. An end device has the possibility to send rejoin-request messages during its communication with the network. This will give the possibility for renewing the security context, changing the DevAddr by the network and changing of the join server. In Figure 5, the packet flow for OTAA activation is shown.

The previous LoRaWAN standard v1.0 [9] specified another way to perform OTAA. We describe the old standard too, as both are currently in use. According to the old standard [9], a join-request message consists of a DevEUI, AppEUI, and a random 2 bytes DevNonce. The join-request is not encrypted, but the MIC is calculated using AppKey. The join-request is accepted by the network server if the integrity check is correct and the DevNonce was not used previously by the end device. If accepted, the network server will reply with a join-accept message. The join-accept message will contain DevAddr and 3 bytes of AppNonce among other communication parameters. AppKey is used by the end node to generate all the rest of session keys, AppSKey and NwkSKey, respectively.

Contrary to OTAA, in case of ABP, all the session keys are saved beforehand. There is no need for activation process and exchange of join-request and join-accept packets.

## 3. Applications and Deployments

This section gives an overview of the current deployments and uses cases that make use of LoRa and LoRaWAN as communication protocol. Several application studies are presented and described in detail.

### 3.1. Applications

LoRaWAN is used for different application types ranging from health and wellbeing monitoring [12,13], agriculture monitoring [14,15,16,17], wireless sensor networks [18,19], traffic monitoring [20], localization [21,22,23], smart city applications [24] up to smart grids and tele-measurements [25,26,27,28]. It is used mainly for non-latency-sensitive applications and for applications where large-scale deployments are needed. According to Adelantado et al [29], LoRaWAN is feasible for use cases such as smart-city applications, metering and logistics (tracking), while it is less feasible for real-time monitoring (unless low scale deployment is used) and not feasible at all for cases such as video surveillance.

LoRaWAN is used for application cases that have communication asymmetry, meaning that the uplink data traffic volume is higher than the downlink data traffic volume. An evaluation of LoRaWAN usage for wireless sensor networks is presented in [18]. Based on a measurement campaign, the reliability of LoRaWAN has been assessed in a city deployment. It was demonstrated that over a distance of 2 km the reliability in terms of packet reception ration (PRR) was 95.5% [18]. Apart from a static deployment of sensors, in some cases, sensor networks might employ mobile sensor nodes too. Thus, the impact of mobility on the performance of LoRaWAN has been studied in [30]. Two key findings regarding the impact of mobility on LoRaWAN have been reported: (i) LoRaWAN is susceptible to mobility; and (ii) the effect of mobility worsens the performance for end nodes in case of bad reception conditions (indoor environments or far from gateway). Packet losses can be as high as 20% for indoor mobile end nodes far from gateways, while for static end nodes at the same distance losses were less than 2%. In outdoor environments, PLR could go up to 45% for a distance of 0.4 miles and speed of 15 miles per hour compared to less than 2% for static end nodes. All the measurements were performed with ADR enabled. This study shows that in general the LoRaWAN ADR scheme cannot cope well with mobility, even though it ensures smooth coverage. In general, it is not able to react fast in response to dynamicity of the network.

In addition to general usage for wireless sensor networks, different health-care apparatus solutions can base their communication on LoRa and LoRaWAN. In [12], LoRaWAN has been used as a communication solution for remote point-of-care screening for urinary tract infection. Authors integrated the bio-fluid sample tester system with LoRaWAN node, to send samples over distance. At the other end, the server collects measurement samples and results are made available for access via a mobile app. LoRaWAN has been demonstrated to be useful for Internet of Medical Things (IoMT) too [12]. In agriculture, LoRa is used for low-power monitoring applications requiring long battery life-time for end nodes that are distributed in wide range fields. In [16], a low power LoRa based underground water monitoring system was presented, while Ilie-Ablachim et al. [17] presented a soil monitoring system that uses LoRaWAN communication.

One of the emerging application domains for LoRaWAN are localization and tracking domains. Different methods for localizations exist, like: time difference of arrival (TDoA), RSSI triangulation, angel of arrival, different fingerprinting algorithms, etc. For fingerprinting algorithms to work, large datasets need to be available. In [21], a large-scale LoRaWAN dataset collected in rural and urban areas has been made available. In addition, authors implemented a simple fingerprinting algorithm, *k* nearest neighbors (*kNN*) [31] fingerprinting, to evaluate the accuracy of localization. Using fingerprinting algorithms, LoRaWAN based localization exhibited a mean error of 398.4 m and median error of 273.03 m, when 11 nearest neighbors were used to estimate the location. In [22], TDoA algorithm was used to estimate the location of the end node in LoRaWAN. The location of gateways was assumed to be known. Further, an extended Kalman filtering was used to process the TDoA taken at different time instants with outliers processing. The proposed algorithm gives an accuracy level lower than 100 m [22] that is quite good for LoRaWAN based localization. Another TDoA based localization system in LoRaWAN public network has been evaluated [23]. Table 2 collects all the studies that deal with different LoRaWAN applications and the studied LoRa and LoRaWAN performance indicators.

### 3.2. Deployments

A performance evaluation of a new technology can be done either by simulations or by empirical measurements. For the latter case, networks need to be deployed. In this area, there exist a number of LoRa and LoRaWAN performance studies based on deployed setups in real environments [18,24,32,33,34,35,36,37,38,39]. Table 3 lists each of the studies that made use of a real LoRaWAN setup to study the network performance. It also includes the performance indicators that were measured. The last column of the table shows an example of a measured result and tries to give a comparison between different deployments for the same parameter usage. Note that the RSSI values shown here consider differences in transmit powers used in each study, in order to make values comparable.

In [32], a coverage study for LoRaWAN was presented. A coverage of up to 15 km using SF12 was found based on empirical measurements. At a distance of 2 km from the gateway, the RSSI value was higher than −90 dBm, showing a good coverage in a city environment for all SFs. In [34], packet losses for SF12 at ~2 km were around 3%, while reliability tests in [18] showed that, for a distance of 1.9 km, only 42% of ACKs from the end node could be received.

In [36], a LoRaWAN deployment for indoor environment was evaluated. The gateway was positioned outside, while measurement points were chosen inside the building. For all measurements points, the packet success rate was above 95%, while the path loss ranged from 97 dB to 146 dB, which brings the signal at the bottom level of the receiver sensitivity (tx power 14 dBm), but still acceptable for good reception. In [37], the indoor propagation performance of LoRaWAN has been studied. The propagation performance measurement results were benchmarked to a number of propagation models (ITU model, multi-wall model and ray tracing model) and it was found that the best model that predicts indoor LoRa propagation is the multi-wall model. Another LoRaWAN indoor deployment was presented in [39], in an industrial environment. The gateway was installed indoor and measurement points included, in addition to indoor positions, also points at the outdoor vicinity of the industrial environment. It was shown that a total industrial indoor area of 34,000 m${}^{2}$ could be covered by single gateway using SF7, while outdoor vicinity area still could be reached using SF12.

## 4. Tools and Methodology

In Section 3.2, several real deployments were presented. All of these deployments include only one gateway and a limited number of end nodes. The measurements at different points were carried out with a single end device at a time. In case of large-scale deployments, interference will be the main cause of packet loss and coverage outage. In all the aforementioned studies, only the communication between a single end device and the network was assessed. The network behavior will become different once many devices will simultaneously start using the same network. However, the deployments are good enough to predict the coverage boundaries for certain SFs, but they cannot be used for network scalability assessment. Simulators and test-beds can be used to make repetitive experiments, assess network performance and check the impact of each transmission parameter (transmit power and SF distribution, ADR mechanism, retransmission schemes, etc.).

### 4.1. System Level Simulators

System level simulators are used to perform reproducible experiments for different systems, considering the impact of all parameters in the overall performance of the system. Such simulators are beneficial for determining the overall network performance when there is no large scale testbed deployed. Using such an approach, LoRaWAN network level performance under certain heavy condition scenarios, such as large number of end nodes or high traffic loads, can be studied. For this, a number of system level simulators have been presented in the literature [24,34,40,41,42,43,44,45,46,47,48,49]. They account for different LoRa physical layer characteristics as well as LoRaWAN MAC behavior. NS3 is one of the most common used network level simulators. Due to this, a number of LoRaWAN simulators are NS3 compatible [40,42,44,48]. On the other hand, there exist other simulators implemented in Python [41] and C++ [24]. However, not all of them are publicly available and open source.

The network simulator in [41] is a simple simulator that can be used to assess the performance of LoRaWANs, however, it has some drawbacks. First, the collision property is only based on the average RSSI difference and relative timing between transmissions, not accounting for the SNR values between transmissions. Moreover, signals received with different SFs are considered purely orthogonal, that is not true under certain power level conditions. The end node parameters (SF and transmit power) can be adjusted based on the distance from the gateway and it offers the possibility to run networks with multiple gateways. However, LoRaSim does not implement any ACK or downlink traffic possibility. An extended version of LoRaSim with downlink support has been presented in [50].

The study performed in [51] determined the Signal-to-Interference (SIR) ratio threshold at which a signal can be correctly decoded under inter-SF interference. The study was based on MATLAB simulations as well as real node measurements of SIR. Based on these figures, the authors of [51] extended the LoRaSim simulator [41] to receive both packets under inter-SF interference condition only when the differences between RSSI is below certain threshold (in this case 6 dB). In addition, they also extended the simulator to account for log-normal channel fading.

Another simulator implementation offers the possibility to use MAC commands, collision based on interference between different SFs as well as multi-GW support, however, it lacks the downlink traffic as well as confirmed uplink traffic [48].

The physical layer model in [40] is based on a LoRa PHY error model that is determined using an extensive set of physical layer simulations in MATLAB. Interference from other signals using different SFs and the same channel is considered by calculating instant SNR values. Contrary to this, the physical layer model in [42] considers only the received power level of the concurrent transmissions and not the overall interference impact. This approach leads to an underestimation of the interference and collision impact in the network. In [40], multi-gateway networks can be simulated. The implementation also includes downlink traffic, ACKs as well as confirmed downlink/uplink traffic. In addition, different SF assignment techniques can be considered: random, fixed or PER based. A drawback of the implementation in [40] is that no MAC commands are implemented. However, this one will not impact the performance evaluation of LoRaWAN, unless ADR impact will be assessed. Contrarily, in [42], MAC commands are implemented. In [42], there are some small flows in implementation that differ from the standard: for example, the MAC at the network side is implemented in the gateway and not in the network server; the end node will wait a random time before transmission (that does not make a real Aloha); and, at the gateway, the downlink traffic is delayed for the next RX-WINDOW if there is uplink traffic being received.

In [52], the authors implemented another ns3 LoRaWAN module that was extended with CSMA features. Other MAC techniques based on listen-before-talk mechanism on top of LoRa were assessed. Kouvelas et al. [53] extended the LoRaWAN module from [48] with p-CSMA module to account for other MAC features in LoRaWAN. Table 4 lists all the open source network level simulators for LoRaWAN and the features that they include.

### 4.2. Testbed Deployments

In addition to the network simulator tools to assess the network performance, LoRaWAN testbeds are another way to assess different network parameters and their impact on performance. Until now, several small scale deployments already have been deployed [18,24,32,33,34,35,36,37,38,39,54,55,56] that include a small number of end nodes. For large scale and massive deployments of test-beds, frameworks and methodologies for easy deployment, control and management are required.

In [57,58], a framework for cloud management of different LPWAN network devices has been presented, where LoRaWAN gateways and network servers are considered too. The control APIs of different network equipment are unified in Lightweight Machine to Machine (LwM2M) APIs using a modular virtual device adapter. The unified APIs make it easy for managing of different network devices from a central cloud-based network operator. Such an approach can be applied to any device type and vendor by only modifying the adapter module that makes the translation of APIs. In addition, the framework can be easily deployed in multiple devices by using docker images.

The study in [59] went a step further in their framework by controlling and managing not only network devices but also end devices. Different parameters of the end devices can be controlled, such as SF used, transmit power, channel bandwidth and FEC code rates. This will make it possible to have controllable and reproducible experiments in LoRaWAN testbeds. Moreover, the network can be controlled by controlling each node parameter individually.

## 5. Physical Layer Performance Evaluation

The research community has given great attention to LoRa and LoRaWAN technological aspects. Numerous studies research the coverage aspects, LoRa resilience to self-interference and other type of interference. This section covers the physical layer performance evaluation studies. It includes studies related to coverage performance and interference impact on communication.

### 5.1. Coverage

LoRa coverage depends on transmission parameters (mainly SFs, transmit power and channel bandwidth used) and environment conditions where the gateways and the end nodes are deployed. Several empirical studies were performed to assess coverage for outdoor and indoor environments as well as indoor-to-outdoor coverage.

In [32], a measurement campaign performed in the city of Oulu in Finland (mostly a flat city) is presented. The gateway was placed in an antenna tower at 24 m above the sea level. Results showed that in the range of 2 km the packet loss ratio (PLR) was 12% while the Received Signal Strength Indicator (RSSI) was above −100 dBm. For ranges from 2 to 5 km, the PLR was 15%; for ranges from 5 to 10 km, 33% of packets were lost; and above this range, 74% of packets were lost. For measurements in open sea area losses were lower, being 31% for ranges 5–15 km and 38% for ranges 15–30 km, respectively. Thus, it is shown that LoRa can achieve good coverage range (up to ~5 km) while packet loss performance can degrade faster in the areas above 5 km. No ADR, repetition or acknowledgement mechanism was considered in this study. Long coverage in such a case comes as a result of terrain configuration. It is expected that in more hilly terrain the coverage would be lower. However, still the coverage distance will be acceptable in terms of some km.

**Figure 6:**
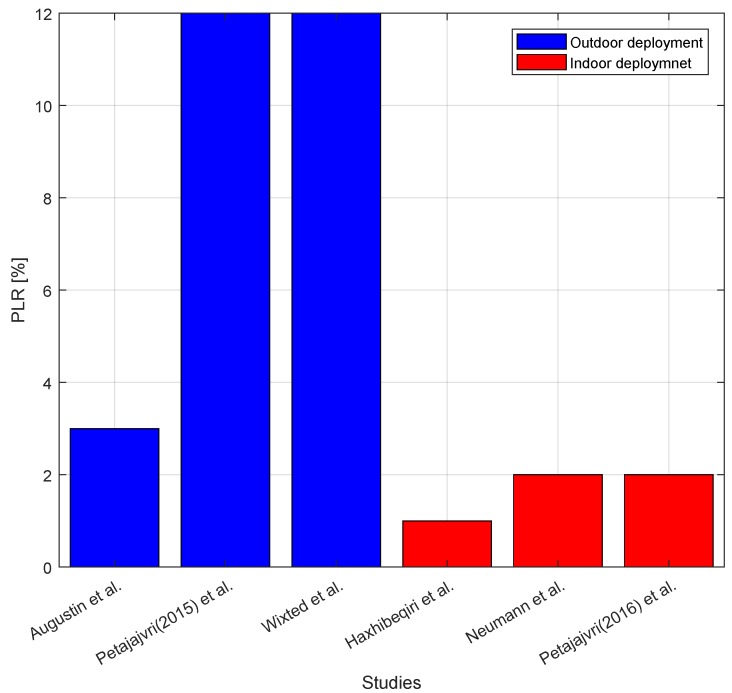
Packet loss ratio for different coverage test using SF12. Distanc from gateway was ~2 km for outdoor measurements and ~60 m for indoor measurements.

Another coverage test was performed in an urban environment with 5–6 floor buildings [33]. LoRa gateway was deployed on top of two store building. The achieved coverage was around 2 km. However, beyond 1.5 km range only SF12 could be used for coverage. Based on such coverage range authors of [33] calculate a rough coverage planning for the city that has 100 km${}^{2}$. With such a conservative coverage range, the whole city can be covered with only 30 gateways. In [34] Lora gateway was deployed on the second floor of a house in a suburban environment. Coverage using SF12 could go up to 2800 m with 20% PLR. The other tested SFs had lower coverage, SF7 having coverage of 650 m with 18% PLR while SF9 having coverage up to 2300 m with 12% PLR. Similarly, in [60] it was shown that in highly built areas LoRa coverage will decrease to less than 1 km for SF10. In Figure 6, the PLR from different studies for the same transmission configuration is shown. In general, it can be concluded that in city environments coverages up to a km can be easily achievable using SF7. For longer coverage that can go even beyond 2 km, higher SFs (SF12) should be used.

In addition to outdoor environments, other authors studied the indoor coverage [35,36,37]. LoRa indoor coverage is sufficiently good, offering RSSI values that are even higher than −70 dBm [37]. Under such conditions, all SFs can be used for communication. In addition to indoor communication, LoRa can be used to offer communication in an industrial area of 34,000 m${}^{2}$ only with SF7 [39]. By using higher SFs the coverage area can be extended even further. For other industrial applications the LoRa radio might be connected to a mobile object or a rotated object. Thus, in [61] an empirical evaluation to account for the impact of Doppler shift and angular velocity in LoRa performance has been presented. It is shown that when the end node moves with a speed of 100 km/h only one third of packets are received correctly. In addition, it is shown that under angular velocities higher than 78 rad/s LoRa communication using SF12 and 125 MHz channel bandwidth becomes unreliable with up to 50% packet losses.

### 5.2. Interference Impact on LoRa

LoRa self-interference plays a determining role in the network scalability. This interference can happen between transmissions using the same SF and different SFs. In addition, as LoRaWAN operates on unlicensed bands, interference from other network technologies should be considered too. To this end, empirical measurements, either in controllable setups or not, are one approach to study interference aspects of physical layer technology. This subsection covers studies related to interference impact on LoRa. Section 5.2.1 includes studies for interference impact from other technologies, while Section 5.2.2 includes studies related to LoRa self-interference impact.

#### 5.2.1. Interference from Other Technologies

In Europe, LoRaWAN uses channels at 868 MHz sub-band that is an unlicensed band. Other technologies such as SigFox, IEEE 802.15.4g, Z-Wave and IO Home Control use the same frequency band. Thus, cross-technology interference (CTI) will impact LoRa based communication and has to be quantified.

In [62], the LoRa’s resilience to CTI by IEEE 802.15.4g was studied. IEEE 802.15.4g uses 34 channels in in the license free band of 868 MHz. The channel bandwidth is 200 KHz wide. The center frequency of each channel is determined by the formula

(3) $$F_{center}=F_{0}+Nr_{Chan}\cdot Chan_{Spacing}$$

where $F_{0}$ is 863.125 MHz, $Nr_{Chan}$ is 0–33 and $Chan_{Spacing}$ is 0.2 MHz. Using this formula, it can be seen that channels 24–28 of IEEE 802.15.4g can overlap with one of the Lora channels. Measurements in [62] were done in a controllable setup where LoRa was set to uses channels centered at 868.3 MHz with different channel bandwidths (125, 250 and 500 KHz), while IEEE 802.15.4g node used channels 24–28 that overlap with one of the mandatory LoRa channels. LoRa is found to be resilient to IEEE 802.15.4g interference. For SF higher than SF9, packet losses are negligible even for an interferer of 16 dB stronger. However, for SF lower than SF9 LoRa gets interfered only when IEEE 802.15.4g node operates in channel 26. In such configuration for strong interferer (+10 dB) PLR can be as high as 90% in case of SF7 [62].

In [63], differences among ultra-narrow band (UNB), Sigfox-like, and wide spread spectrum, LoRa-like technologies in terms of range and resistance against interference were studied. Network topology for simulation consist of two co-located UNB and wide spread spectrum networks in an area of 1000 by 1000 m. Each of the network has 1000 active devices spread in the area and only one gateway. By means of network level simulator, authors of [63] showed that the UNB networks cope well with wide spread spectrum network interference, while the reverse is valid for the vice-versa case. End nodes far from gateway will have more severe performance degradation for LoRa-like technologies under UNB interference. It means that, in the case of coexistence of LoRa and Sigfox, LoRa network will have higher losses due to SigFox interference, being up to 50% for LoRa end nodes 100 m away from gateways.

In [64], impact of SigFox, Z-Wave and IO Home Control on LoRa has been measured by means of a controllable setup in an RF-shielded environment. The controllable parameters during measurements were: power and time difference between the main transmission and the interferer, and SF used by LoRa transmitter. SigFox has the highest impact of all three technologies with LoRa packet losses being up to 28% when the interference starts during LoRa preamble and header time. For the same configuration, losses can be up to maximum of 17% in case of Z-wave. The other time differences, during payload time, show lower losses especially for Z-Wave and IO Home Control technologies due to their short time on air. The high losses caused by SigFox impact are related to its long time-on-air that could interfere the whole LoRa packet even when SF12 is used.

The study in [65] presents a measurement campaign for 868 MHz band signal activity for different environments in the city of Aalborg, Denmark. This study helps to determine the interference probability from other technologies that are currently deployed at 868 MHz band. It is shown that there is 33.7% and 0.9% interference probability in the 868.0–868.6 MHz sub-band and 869.4–869.65 MHz, respectively, in a shopping area. For a business park area, these figures are 22.8% and 58.2% for respective sub-bands. This measurement campaign revealed that in a business area chances are quite high that LoRa communication can get interfered by other technologies. For other environments (hospital complex, industrial area and residential area), interference chances are always lower than 5% for both sub-bands. Thus, in such an environment, LoRa can be a feasible technology to be used for different applications, due to low level of signal activity in LoRa bands [65].

#### 5.2.2. LoRa Self-Interference

In addition to interference from other technologies, LoRa communication will experience interference also from other LoRa based co-located networks. Inter-SF interference will impact LoRa communication even for end nodes that belong to the same network. Thus, LoRa self-interference was studied in several research papers [45,47,51,63,66,67,68].

In [63], self-interference of LoRa-like network was studied. The simulated network topology consisted of two gateways that belongs to two different networks and are located at the opposite corners of a square with dimensions 1000 m × 1000 m. Each network has 60 nodes distributed inside the square area. Results show that end nodes at distance 1000 m away from the gateway reach a PLR of up to 50% as a result of interference [63]. Even though SFs are thought to be orthogonal in theory, due to orthogonality imperfections they can interfere between each other.

In [51,66], SFs imperfection orthogonality impact on self-interference has been studied. It is shown that inter-SF interference deteriorate performance of the network even under low traffic load, especially for higher SFs (SF10–SF12). The impact of log-normal channel fading will have negative impact only in single gateway deployment scenarios, having up to 15% lower Date Extraction Rate (DER) [51]. On the other hand, for multi-gateway deployment, fading impact will be negligible. Due to gateway diversity, it is possible for a packet to get received by different gateways decreasing the impact of fading [51]. Another empirical study regarding the LoRa self-interference was presented in [67], where similar results as in [51] were observed.

In [45,47], the collision behavior at link level between two different LoRa packets was studied. Collision behavior depends on carrier frequency, spreading factor, received power and time difference between interfering transmissions. In [45] authors proved that LoRa experiences capture effect, meaning that the stronger signal can suppress the lower one, and can be decoded correctly at the receiver. Based on this outcome, in [45] authors proposed two mechanism to decrease the LoRa self-interference by using multi-gateway deployments and directional antennas for end nodes. The first solution is seen to be beneficial even for removing negative effects of fading channels [51]. By using directional antennas for end nodes, the received signal becomes stronger as well as the interference from other interferer network lowers. It is shown that, when directional antennas with 8 dBi gain are used, DER is improved by 0.06 compared to cases when omni-directional antennas are used [45].

## 6. MAC Layer Performance Evaluation

Section 5 reviews studies related to physical layer performance, while this section reviews studies that are related to LoRaWAN MAC layer performance. First, it makes a detailed review of the LoRaWAN MAC models that are used to assess network performance. Then, power usage evaluation and security mechanisms and security vulnerabilities are covered.

### 6.1. LoRaWAN Network Models

Network wide performance is hard to be assessed by measurements in real networks as it is nearly impossible to have reproducible experiments. Moreover, there is still no large-scale LoRaWAN test-bed. Thus, models about self-interference, channel access techniques and joint-procedure can help to assess LoRaWAN performance. However, as near to reality the model is, the better performance assessment is. For this, different approaches are taken by researchers. There exist some mathematical models that consider only medium access characteristics while other researchers based their model on empirical studies of the physical layer performance. The following subsections review both of these approaches and models.

#### 6.1.1. Mathematical Models

Several analytical network models for LoRaWAN performance are described in the literature [25,34,69,70,71,72,73,74]. They differ based on what type of SF interference is considered, if the channel fading is considered [25,73] or if other technology interference is considered too [25]. On the other hand, the LoRa modulation mathematical model was presented in [4]. It was shown analytical that, in comparison to FSK modulation, LoRa modulation performs better in case of frequency selective fading.

At the beginning of LoRaWAN research, the channel access model was assumed to be Aloha. In [34], authors modeled the channel access in LoRaWAN as Aloha based and assessed via the simulations the network capacity. However, their assumption that, if the transmission time of two packets overlaps, none of the packets will be received correctly is proven to be wrong by other empirical studies [47,75]. Thus, the network capacity of LoRaWAN was under-determined by over-determining the collision ratio.

A mathematical model based on Aloha is shown in [69]. Authors of [69] considered only MAC layer characteristics including acknowledgments and retransmissions. Frame generation by end nodes is modeled as Poisson distribution, while model considers uplink packet probability as well as downlink ACK probability for a successful transmission. The proposed model offers the possibility to find the PER under certain traffic load. It is shown that PER is 50% lower when retransmissions are considered compared to the case without retransmissions. In [70], the model has been extended to include capture effect with each co-SF interferer separately, while it misses the impact of channel fading.

In [71], a system model for uplink communication in single gateway deployments is shown using stochastic geometry modeling. For the outage probability of a desired signal in uplink two conditions are considered: if the signal is above a certain SNR threshold and if the signal is at least 6 dB stronger than the dominant co-SF interferer. This study later was complemented by considering also the cumulative co-SF interference and inter-SF interference alone, as well as cumulative impact of both [73]. Compared to [70], studies in [71,73] consider also impact of channel fading modeled as Rayleigh fading.

Authors of [72] extended the model presented in [71] for the case when gateway is equipped with multiple antennas. Analytically, it is shown that by employing two and four antennas at gateway the coverage probability is increased by 1.5 and 1.97 times, respectively, compared to single antenna case [71]. In addition, the impact of message replication in coverage probability is shown, being utilized better for low density networks. In higher density networks, it causes more problems (higher collision probability) than that can increases the coverage probability.

Another analytical model is presented in [76] that considers the impact of duty cycling in latency and collision performance. The SF orthogonality assumption makes it not realistic in terms of collision performance evaluation, however, it is one of the first models that analysis the latency behavior under duty cycling regulation constraints.

In [74], the joint time-frequency interference is considered as a two-dimensional plane to account for the overlap of transmitted packets in both dimensions. The generated mathematical model considers the capture effect too, meaning that even under collision the packet with better received SINR can get through.

In [77], a Markovian chain model to assess the performance of the join procedure in LoRaWAN was presented. Based on generated model, the delay performance and energy consumption of join procedure are assessed. It is shown that the joining delay is impacted by the number of channels and sub-bands used, being around 50 s when all channels are used [77]. The percentage of inactivated nodes has a negative impact in the activation delay by increasing the delay linearly. Table 5 lists all the models together with the network features that they consider.

#### 6.1.2. Empirical Based

Apart from theoretical approach, other system models are based on empirical approach by doing controllable measurements with certain number of nodes and generating models based on outcomes.

In [75], authors performed measurements for co-SF interference in a controllable setup, where received signal strength and time difference between interferer and interfered signal were changed. Based on the measurements, it is found that the most fragile parts of a packet are the preamble and physical header with main three outcomes [75]:

- If the interferer starts after the preamble and the RSSI from the interferer is at the same level or lower than the interfered transmission, then the interfered transmission will be received correctly.
- If the interferer starts after the end of the preamble and the header time and has a higher RSSI at the receiver, then the first transmission will be received with the wrong payload CRC.
- If the last six symbols of the transmitter preamble are received correctly, the receiver can synchronize with the transmitter.

Similar results were achieved by Ferrari et al [78] accounting for the pseudo-orthogonality of SFs.

Based on these outcomes a system model was created to assess scalability of the system. It is shown that for multi-SF single gateway deployments LoRaWAN can have three times lower losses compared to Aloha for 1000 end nodes per gateway. As the model does not consider the inter-SF interference (SF orthogonality is assumed), this can be seen as top border of performance, as in this case the inter-SF interference is under determined. Similar approach was followed in [47], where LoRa capture effect was determined by empirical measurements. Another empirical based system level model has been presented in [68] that considers inter-SF interference. In addition, authors used a corrected Okumura–Hata path loss model (based on coverage measurements) as well as log-normal shadowing effects in their model.

Differently from empirical approaches, in [40], LoRa interference model is constructed based on extensive complex base band bit error rate simulations. The interference model is combined with LoRaWAN MAC protocol to design system level model for LoRaWAN. A packet is received correctly if the SINR level (interference from other SFs are considered too) is above the receiving threshold for that SF.

**Figure 7:**
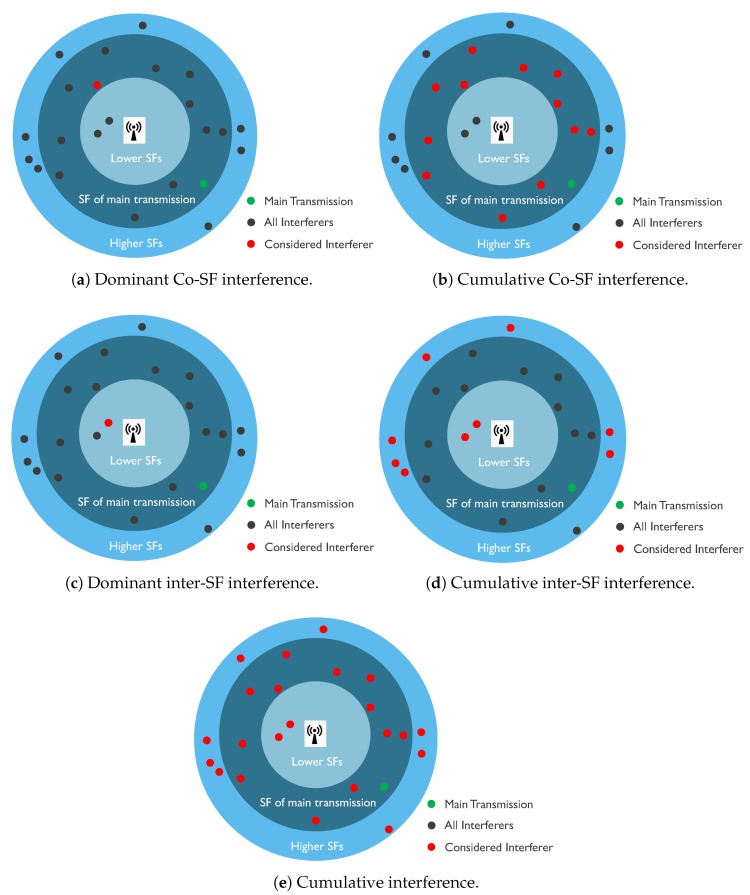
Interference scenarios that are taken into account in LoRaWAN models.

Table 5 lists all the models together with the interference scenarios that they consider. In Figure 7, different interference scenarios are shown based on what type of interference are considered. In Figure 7a, the case where interferer using same SF with highest signal strength reception (red end node) in the gateway is shown. Figure 7b shows the case where all interferers using the same SF, no matter their signal strength at receiver (red end nodes) in the gateway are considered in the model. Figure 7c,d shows the cases when dominant and cumulative inter-SF, respectively, interferer are considered for the interference model. Figure 7e shows the case where all type of LoRa self-interference are considered.

### 6.2. Power Usage

Device power consumption is crucial for proper operation, as LoRaWAN is used for application employing constrained devices. Thus, it is needed to model the power usage profile of LoRaWAN end node and to determine the battery lifetime. The presented power consumption values are based on empirical measurements done with certain end nodes [35,79,80]. They include all the phases that an end node will transit during its transmission phase: sleeping time, wake-up time, transmission time, listening in the first receive window and receiving phase.

In [79], a power profile modeling of LoRaWAN end node is shown. The model considered the impact on power consumption of residual bit error rate (BER) after the bit error correction is done using FECs. In addition, collision probability impact is considered too. It models both type of transmissions: acknowledged and unacknowledged ones. Evaluation is done based on the values for current consumption by mDot end node at each of the transmission phase. It is shown that, by sending a packet (51 bytes) every 10 min, battery life can be up to one year for SF7–SF10, for a battery capacity of 2400 mAh. On the other hand, for SF12 and SF11, the battery lifetime will be lower, 0.5 and 0.8 years, respectively [79]. For less frequent transmission, lifetime will extend. It is shown also that the power consumption in unacknowledged uplink traffic case is higher than the case when the acknowledgment is received in the first receive window. Such an observation is not intuitive. In the latter case the second receive window as well as the phase between the first receive window and the second receive window does not happen. This makes possible for the node to go to sleep right after the reception of ACK packet. In the former case, as there is no ACK, end node still needs to open both receive windows and to pass the phase between two receive windows. This will introduce higher power consumption than the reception of small ACK in RW1.

In [80], authors compared the battery lifetime for different LoRaWAN end device classes. First, voltage and current drain measurements for each end device class are done. Based on the measurements, the results are benchmarked with the end node data-sheets. Then, the battery capacity for a period over 10 years for different packet lengths, transmission periods, SFs and different device classes are calculated. In all cases, an-acknowledged traffic is supposed.

A LoRa energy efficiency study is presented in [81]. It is shown that energy consumption is increased faster by increasing the SF while keeping the transmit power the same. Thus, more desirable for ADR mechanism is, first, to increase the transmit power for increased range before the SF is increased. In [82], the benefit of using ADR scheme and long packet length in LoRa energy performance is shown. The ADR mechanism reduces the energy consumption five times per payload byte. Energy consumption for payload byte decreases also for longer data packets due to lower header overhead [82]. However, this cannot be true in congested network where longer packets are prone to higher error rates and need to be retransmitted. In [83], power consumption profile for Semtech SX1272 chip is modeled. The average battery lifetime is calculated to be 1–5 years for sensing interval of 1–5 min, respectively. If energy harvesting from renewable energy sources is used, battery lifetime can go from 4 to 12 years for the same sensing interval range.

In Table 6, studies that deal with power consumption modeling are collected by showing what type of traffic is considered.

### 6.3. Security

For every IoT application, security is a fragile part. Thus, security issues for each newly IoT technology have to be investigated from the early stage of deployment. Even though LoRaWAN is rather new IoT technology, in the first standard release [9], basic security principles were founded. With the newest standard release [11], security is improved by introducing new method for security key generation for OTAA, and re-joining mechanisms. In Section 2.2.2, we give a detailed description of security mechanism in LoRaWAN for both standards. Here, we describe the vulnerabilities in both standards as both are being used.

Numerous research works showed the security vulnerabilities in LoRaWAN v1.0 [84,85,86,87,88,89]. Considered security vulnerabilities include the replay attack of join-request packet for OTAA, data packet replay attack in case of ABP, eavesdropping of data packets in ABP case, bit flipping attack between network server and application server, ACK spoofing, etc.

Replay attacks (join-requests packets in OTAA or data packets in ABP case) can end up in Denial of Service (DoS) for certain end nodes. In OTAA case, DoS attacks can happen if the network server will decide to exclude the device from the network due to wrong join-request packet. There are two cases: a join-request packet sent by a trusted node can be mistakenly categorized as a replay attack (due to system generation of DevNonce in LoRaWAN v1.0) or there will be a real replay attack. For LoRaWAN v1.1, the DevNonce is a counter number and no longer random. This will ensure that end node will not produce the same DevNonce for different join-request messages.

LoraWAN v1.0 standard specifies that if the uplink or downlink frame counters differ from the last counter at the receiver side, and the difference is greater than a specific value MAX_FNCT_GAP, the subsequent data packets from that node will be discarded. As in ABP end node uses static NetSKey and AppSKey, a data packet replay attacks can lead to DoS due to this feature of the standard. What a malicious node can do is to intercept an uplink data packet and wait for end node reset or counter overflow. Once the counter is reset, the malicious node can replay a packet with higher counter that will lead to higher MAX_FNCT_GAP and thus to DoS for that node from the network server. The replayed packet is still valid as the NetSKey and AppSKey did not change after the node reset. This vulnerability exists only for LoRaWAN standard v1.0, as in LoRaWAN standard v1.1 the MAX_FNCT_GAP concept is removed and even in case of replay attack the DoS will not happen.

Eavesdropping of two different packets with the same counter will lead to decryption of data packet payload by the malicious node. This can happen once the end nodes is reset, or the packet counter is overflowed. As data packets payload are encrypted using AES in counter mode, and, moreover, the packet counter value is used as input, decrypting the sniffed data packets using the same counter is trivial. This is shown in [87]. In the case of OTAA with the newest version of LoRaWAN v1.1 [11], counters are not allowed to be used during the same activation session. After every reset or before the counter overflow, end node must perform a re-joining process [11]. However, the vulnerability is still valid for ABP case.

The network provider is separated from the application provider in LoRaWAN networks. Message integrity check (MIC) of the data payload and header information is done at the network server, while the payload decryption using AppSKey is terminated at application server. In between the network server and application server, there is no means of message integrity check. This will expose the data packets to bit flipping attacks, and they can go undetected. This vulnerability exists in both standards.

In [90], security analysis of join procedure in LoRaWAN is studied. The current implementation of DevNonce random generation in the WiMOD SK-iM880A is done by performing 16 read operations of the least significant bit (LSB) of the register RegRssiWideBand. Under jamming conditions, this register might be overflowed, resulting in the same value all the time. Thus, in [90], authors evaluated the probability for the end node to generate the same DevNonce during an interval of generated join-requests. To avoid the DoS mistaken cases (for LoRaWAN v1.0), authors of [90] proposed using a sequential number as DevNonce, by keeping track of the last DevNonce used at both ends. This feature is already included in specifications for LoRaWAN v1.1 [11]. However, this approach might be vulnerable to attackers that can easily predict the next DevNonce. In [91], the join-request packets RSSI values are tracked by the network server in addition to previous DevNonce used. If a join-request packet with the same DevNonce is received, the RSSI value is checked to categorize if the join-request packet is replayed by an attacker or not. However, for mobile nodes such an approach does not work as the RSSI value will change by node position. In such a case, a packet send by a mobile node mistakenly will be categorized as a replay attack. In [92], a hybrid cryptosystem to enhance LoRaWAN v1.0 join procedure security is presented. An asynchronous cryptosystem is implemented in addition to current synchronous cryptosystem. This means that end node will request the network server public key before it sends the join-request packet. Then the end node will first transmit the AppKey encrypted join-request packet and NS public key encrypted of the AppKey information. The network server will decrypt the join-request and reply with the join-accept packet [92]. However, even though this method is secure and avoids replay attacks, it requires additional handshakes in the join procedure that will decrease the bandwidth for the end node. Bandwidth shortage will become even more severe for bands and regions where duty cycle is required.

Another approach to prevent replay attacks is a token-based approach shown in [93]. The join-request packet is masked by a unique token using an XOR operation. Thus, even if join-request packets are intercepted by an attacker, they cannot be used as they are masked with different tokens. Tokens are generated from the previous NetSKey. However, such a method is still vulnerable in cases when end node forgets the previous NetSKey due to reset or any other action. To avoid such an issue, in [94], join-request packets are divided in initial ones and non-initial ones. An initial join request is sent in case when end device does not have any previous NetSKey saved. For non-initial join-request packets, MIC is generated using previous NetSKey. To alleviate the data packet replay attacks, in ABP the NetSKey and AppSKey should not be static. There should be a mechanism that changes the keys every time the node is reset [87]. Such an approach is now adopted in the newest standard where rejoin procedure is introduced every time the node is reset or before the overflow of counters. Table 7 collects all the research and their studied security aspects together with the offered solution.

## 7. LoRaWAN Improvements

Section 3.2 and Section 5.1 show that LoRaWAN coverage can go up to several kilometers. This involves that single gateway can cover many end devices. Researchers propose several LoRaWAN improvements to deal with network scalability and traffic reliability. This section details the studies related to ADR improvements for improved network scalability as well as different synchronization and traffic scheduling approaches.

### 7.1. Network Scalability Assessment and Improvement Methods

Several studies reported about LoRaWAN network scalability concerns [32,40,50,75,97,98], especially for the downlink traffic impact on scalability. By means of network simulations, it is shown that LoRaWAN can end in a deadlock stage if certain scalability issues are not addressed by time. Thus, several ways how SFs and transmit powers are distributed, as well as improvements to ADR mechanism are proposed.

One of the first studies on scalability analysis is shown in [99]. The capacity of single gateway deployment under perfect synchronization and number of end devices for optimal Aloha case are derived. According to channel attenuation model shown in [32], it is shown that less than 2% of total end devices will use SF12. In addition, it is demonstrated that end devices far away from gateway have lower throughput compared to those nearby due to near-far problem. This means that packets from far nodes will be collided by the packets from nearby end nodes, due to better reception conditions. Due to this, authors of [100] presented an algorithm for assigning SFs and power levels to end nodes. First, the fraction of end nodes using certain SF inside one cell is optimized by minimizing the probability of having inter-SF collision. The second step is to find the optimal distribution of SFs and power levels. All the end nodes are sorted according to their path loss and are divided in a number of groups as there are channels available in the network. Then, in each group, nodes with lower path loss will get higher SF compared to those with higher path loss. This assignment will follow the optimized fraction of SFs found in the first step. It is proved that using such an algorithm PER is decreased by 50% for nodes at the cell edge in a scenario with 1 end node per 1000 m${}^{2}$ transmitting every 10 min.

The impact of SF distribution among end nodes in packet success probability (PSP) is appreciated in [101]. In [101], the regions of SF distribution are determined with the intention to increase the average PSP of the network. When assigning SFs to end nodes two conditions are considered: (i) the received power from that node in gateway should exceed the receiver sensitivity threshold for the assigned SF; and (ii) SIR for that node must also exceed the correctly decoded SIR threshold for that SF. An optimization problem for assigning SFs is formulated, where average network PSP is maximized with respect to region distances covered by each SF. Lim and Han [101] demonstrated that, when numerous end devices are present in the network, it is reasonable to increase the lower SF region by increasing the probability for Condition (ii) and lower the probability for Condition (i). It is shown that the proposed solution outperforms the equal-interval and equal-area based SF distribution schemes in terms of average network PSP [101]. Two new algorithms for SF distribution in a cell are proposed in [102]. The first one, ExpLoRa-SF, assigns SFs to end nodes by equalizing the number of end nodes using different SFs. To this end, the first group of end nodes that are nearer to gateway based on their RSSI values will get assigned lowest SF and so on until the last group of end nodes. The second proposed scheme, ExpLoRa-AT, tends to equalize transmission times between different end nodes by distributing different SFs. The second approach will optimize the usage of different SFs over time, achieving better network performance. However, the first approach will achieve the same in case when all end nodes will have the same traffic patterns as well as same packet lengths.

In addition to SFs and transmit power assignment algorithms, other improvements in ADR mechanism are proposed in [46,103,104]. In [103], the performance of ADR mechanism [11] in terms of the average converge time is shown. The convergence time depends on network size (200 and 3000 min for networks with 100 and 4000 nodes, respectively) and channel conditions [103]. Interestingly, the highly channel variations will decrease ADR convergence time for large networks (4000 nodes). This is explained with the fact that high channel variations introduce randomness in RSSI values, increasing the impact of capture effect. By optimizing different parameters of the ADR mechanism (ADR_ACK_DELAY and ADR_ACK_LIMIT), authors of [103] showed that only the ADR_ACK_DELAY decrease will show an improvement in ADR convergence time. By reducing the ADR_ACK_DELAY the duration of each individual step to increase transmit power and SF is shortened, speeding up the convergence time.

In general, main factors that cause a packet to be lost are interference, gateway receiver saturation and network outage. It was exhibited that for highly congested LoRaWANs the main factor is the receiver congestion rather than network outage [98]. Thus, in such cases, ADR algorithms or retransmissions mechanisms will not improve the performance, in opposite they will worsen it further.

Authors of [46] proposed an improvement in the current ADR mechanism implemented at the network server side in order to increase network performance. Instead of considering the maximum SNR value from a number of uplink packets in the past, authors proposed to take the average SNR of that window. This improvement helps to alleviate the impact of variable channel conditions. The proposed ADR mechanism achieves at least 30% better packet deliver ratio compared to basic one in cases or moderate variable channels.

Another ADR algorithm that maximizes the data extraction rate (DER) of end nodes was presented in [104]. Authors proved that the fairness index between end nodes using Aloha will decrease by increasing the number of end nodes in the network. This will favor transmissions from end nodes that are closer to gateways compared to far away end nodes. Proposed ADR algorithm in [104] distributes the SFs and transmit powers to end nodes by maximizing the DER fairness index among the end nodes. The implementation is tested in LoRaSim simulator [41].

The impact of downlink traffic in the network performance was studied in [40,50,97,98] and was proved to be deteriorating for the uplink traffic performance in case of large number of end nodes. In [97], it is shown that, by increasing the ratio of high priority end nodes (asking for confirmed uplink traffic), the total network throughput is decreased by worsening the throughput for low priority end devices. Same relation is found even with the number of retransmissions per packet. In [40], it is demonstrated that the number of missed receive windows will increase with the increase of uplink traffic that requires downlink confirmation due to gateway duty cycle.

### 7.2. Synchronization and Scheduling for LoRaWAN

ADR mechanism and different SFs and transmit power distribution will improve network scalability. However, still the main network scalability bottleneck is usage of Aloha based MAC protocol. In addition, the half-duplex operation of gateways as well as duty cycle limitation increased the negative impact on network scalability. To this end, other solutions to coordinate transmissions are being studied. Such solutions rely on low-power time synchronization and low-overhead traffic scheduling mechanisms.

In [105], a lightweight scheduling mechanism to specify the allowed SFs and transmit powers at certain channels is introduced. The time is divided into frames and each frame is divided into *I* subframes, where *I* is the number of channels used. Each subframe starts with a beacon that includes the information such as allowed SFs and RSSI values for each communication channel. In the second part of subframe, end nodes can accesses channels using Aloha in order to transmit their uplink data [105]. The benefit of this approach is that the capture effect is reduced as well as inter-SF collisions are reduced as the end node will select the minimal SF and transmit power that reduce the negative impact to other end devices. The only constraint is that the uplink traffic should not interfere with beaconing traffic. However, the improvements in reliability compared to LoRaWAN are not that high, in the range of 4–5% fewer losses for 3500 end nodes in multi-gateway deployment.

Another synchronization scheme for downlink transmissions is presented in [106]. The synchronization is based on beacons that are broadcasted by gateways and include the DevAddr of the end nodes for which the network has downlink packets. After the beacon transmission, the beacon window is divided in the downlink part and uplink part. In the downlink part, the end nodes will poll for the downlink packets sequentially based on the DevAddr order appeared in the beacon. In the uplink part of beacon window, the uplink communication happens like for normal Class A devices [106]. This approach will offer possibilities for downlink traffic as in Class B devices, while the power consumption will be less than in Class B devices as the downlink traffic is polled based. There is no need for end device to open receive windows at each beaconing period, decreasing the power consumption compared to Class B end devices. However, the uplink traffic performance will not be enhanced as end nodes still will use Aloha based channel access.

Time synchronization of end devices with back-end is crucial for correct system functioning in case of tele-measurement applications in different domains of industry. In [107], a low power time synchronization mechanism for Class A end devices is presented. The time synchronization of end device happens a posteriori, after the event payload has been sent to the network. The end node will receive an ACK packet from the back-end after the main transmission. As a last step, end node will send a second packet that will inform the back-end with the time when the first packet was sent and when ACK was received according to the unsynchronized end node time. At the back-end side the uncertainty of the time-stamp can be calculated and the actual time of the event can be deducted. This mechanism requires low power as it asks only for another short packet to inform back-end for two timestamps. However, such a mechanism cannot function under heavy load, as if any of the packets (ACK or timestamp packet) are lost the system will malfunction. Moreover, the actual time is not known at end node but can be calculated only at the back-end. This implies that such a method cannot be used for traffic scheduling at proper time.

In [108], a low-overhead synchronization and scheduling mechanism is proposed. In such a deployment, the time is divided into time slots, where time slots are maintained by the scheduling entity in the network server. The synchronization mechanism consists of end node asking for the synchronization and scheduling of time slots. The synchronization request can happen in band or in a separate channel reserved for synchronization traffic only. The synchronization and scheduling entity will reply with the current time slot, current time offset in the current time slot, as well as all the future time slots where end node can transmit. The information about scheduled time slots is compressed using probabilistic data structure, making it possible to fit in one packet payload. Based on the reply, the end node can determine the current time and future time slots when it can transmit. Synchronization can happen in-frequently and considers clock drifts of end node. It is shown that PDR of synchronized network is increased by 30% for networks using SF12, using practically the full network capacity. Authors of [108] showed only implementation for single SF deployments. For multi-SF deployments, SNR based scheduling need to be performed. Another approach is to use slotted-Aloha for LoRaWAN [109]. An ACK based time synchronization algorithm is proposed in [109]. It is shown that, by using slotted-Aloha, collision probability is decreased three times compared to pure Aloha.

## 8. Extensions

Researchers are not concentrated only on LoRaWAN performance studies, how different parameter adjustment can improve performance or in improving algorithms that increase security and reliability. They look also into other MAC solutions on top of LoRa, extensions of LoRaWAN stack with support for IP communication, other network topologies as well as multi-technology networks. This section reviews such works that are already published.

### 8.1. New MAC Design for LoRa

Other MAC designs that alleviate the bottlenecks of Aloha can be used on top of LoRa. For example, if CSMA based MAC protocols are included, the duty cycle can be avoided, increasing thus the throughput. In [52], authors showed that by using CSMA the packet delivery ratio is increased by seven times in the case of 10,000 nodes compared to normal Aloha based MAC. In such a case, the PDR of CSMA based MAC is more than 75%, while in case of Aloha-based LoRaWAN MAC the PDR is only 10%. At the same time, the energy consumption per node is decreased. In the case of Aloha-based LoRaWAN MAC, much energy is wasted in collisions. Another comparison study between Aloha-based and p-CSMA based MAC protocols for LoRaWAN is presented in [53].

A time-power multiplexing approach for MAC protocol on top of LoRa was proposed in [110] to improve downlink traffic scalability. The first proposition is to decouple the uplink and downlink traffic. Both receive windows will use only high power (27 dBm) high duty cycle (10%) channel (869.525 MHz) for downlink traffic, while the rest of channels are used only for uplink. This will overcome the collision possibility between downlink and uplink traffic. Secondly, the transmission in downlink is multiplexed in time and power, by sending simultaneously multiple packets using different SFs as well as different transmit powers [110]. This approach shows that the number of retransmissions of confirmed uplink packets is decreased. At the same time, the uplink throughput is increased even for non-confirmed traffic, as they benefit from elimination of collision possibility between UL and DL traffic.

In the case of LoRa mobile nodes, they can communicate with multiple gateways in the area. However, due to long range coverage, end nodes that are covered by different gateways can impact each other traffic due to hidden terminal problem. To avoid the hidden terminal problem, a MAC protocol on top of LoRa was studied in [111,112]. All the nodes are always in the listening state. When they want to transmit, they will send a Request-to-Sent (RTS) message. Multiple gateways will reply with Clear-to-Sent (CTS) but due to capture effect one of the CTS will succeed. Both RTS and CTS messages will include the reservation time for the channel. Before actual data transmission, end node will send another Control CTS (CCTS) to tell to which gateway it will transmit, in order for other gateways to continue serving other nodes. At the end, the data communication happens [112]. This MAC protocol improves the data delivery ration by decreasing the impact of hidden terminal effect.

### 8.2. IPv6 over LoRaWAN

In the current implementation of LoRaWAN protocol stack the application layer resides on top of MAC layer. However, if the end nodes need to be accessed from the Internet, then the LoRaWAN end nodes should become IP compatible. Several standards for IPv6 communication over constrained devices and networks exists, such as adaptation layers (6LowPAN [113], 6Lo [114], and 6TiSCH [115]), header compression techniques (LOWPAN-IPHC [116], LOWPAN-NHC [116], and SCHC [117]) and application layer protocols for constrained devices (CoAP [118]).

Recently, solutions for IPv6 communication over LoRa and LoRaWAN are being studied [119,120,121,122]. In [119], feasibility usage of different existing techniques for enabling IPv6 over constrained devices in LoRaWAN have been studied. The Static Context Header Compression (SCHC) is presented as one of the most feasible candidate for LoRaWAN. It provides header compression for CoAP, UDP and IP as well as fragmentation possibilities. In LPWAN networks, the communication context is quite static, being that end node will communicate with a foreknown server and will use certain services. Thus, in SCHC, the static context is composed of a set of rules that defines how to compress different headers: CoAP, UDP and IPv6. In [120], SCHC was used to compress headers for IPv6 over LoRaWAN for an Internet-to-Vehicle (IoV) application. In [121], the 6LowPAN adaptation layer and IP Header Compression (IPHC) together with fragmentation possibility were used to enable IPv6 communication for LoRaWAN devices. To this end, in static context (where end devices are registered by personalization) the source IP can be removed as there is a unique mapping between DevAddr and source IPv6. In the case of dynamic context (activation over the air), none of the IP addresses (source and destination) can be removed. No UDP header compression is used too.

In [122], Contiki protocol stack was ported to LoRa based node and the IPv6 communication was enabled. The 6LoWPAN adaptation layer as well as radio duty cycle layer are used to comply with duty cycling rules.

### 8.3. LoRa Multihop Networks

Current star topology of LoRaWAN cannot be exploited in every type of application. For end nodes that are located deep indoors or experience bad receiver quality from gateways, multi-hop communication can be a solution. To this end, LoRa multi-hop network topologies are considered as a method to boost up performance especially for such end nodes [19,54,123,124,125]. In other studies, the LoRaWAN end nodes were extended with relying capabilities [126].

Lee and Ke [19] focused in mesh communication for LoRa nodes by introducing a system architecture where the gateway is the central sink. Gateway will start beaconing to advertise its presence. Once a node asks to join the network, the gateway will stop transmitting beacons. Rather, gateway will poll periodically the joined node for the information and these packets will be used by other end nodes as beacons. The other nodes that can hear polling packets from gateway or replying packets from the joined nodes they can ask to join network to certain nodes. The nodes can accept or refuse the join request. If they accept, they inform the gateway for all their “children”. Thus, gateway will have the general network view and will poll each node in the network periodically. It is shown that in such a network topology the PDR for end nodes far away from gateway will improve, for some nodes from 0% to 91% [19]. The tested setup employed only 19 nodes in total deployed indoor and outdoor in an area of 800 m by 600 m [19]. Even that it is seen as a good solution for deep indoor end nodes, for larger deployments network scalability of such a solution might be an issue as each of the nodes has to be polled by the gateway. Moreover, accounting for radio duty cycle and usage of different SFs, the solution might end up using single SF that will decrease the network capacity. If multiple SFs are used in different hops, network can be fragmented.

Another LoRa based multi-hop network was presented in [123]. In this case, traffic flooding in the network is used, by using concurrent transmission (CT) protocol. As LoRa exhibits capture effect, this will help in reception of the highest-power packet at each relay hop. Authors of [123] demonstrated the impact of the power offset, timing offset as well as carrier frequency offset in reception of one of the concurrent transmissions. Based on this empirical evaluation, they proposed a new transmission method, offset-CT, where each node in the relay hop will delay the retransmission by a randomly generated time offset between 0 and $T_{s}$, $T_{s}$ being the symbol time. They showed that the offset-CT improves the packet reception ratio (PRR) from 51% to 77% in case of relay groups with four nodes in a four-hop network topology. Same observations are valid for this solution too, only single SF can be used, otherwise network can end up being fragmented.

In [54], a multi-hop MAC protocol on top of LoRa, LoRaBlink, was proposed. A time slotted channel access scheme is used where time slots are organized in frames. Each time slot frame is divided in beaconing slots and data slots. In the beaconing slots, the first slot is used by sink to transmit its beacon, the second slot is used by the second group of end nodes to transmit their beacons and so on until the last node. The number of beacon slots determines the network depth. For data communication, the leaf node will transmit the uplink packet first, then the next hop will transmit concurrently the data packet. Due to capture effect that LoRa exhibits, one of the packets will succeed and will reach the sink. However, this approach has a drawback if multiple SFs will be used in the network. In such a case, end node must be able to listen simultaneously for multiple SFs and in multiple channels, which will increase the end node price. Otherwise, for single SF and single channel deployment approach network throughput will decrease.

In all the studies published until now for multi-hop network topologies, radio duty cycling is not considered. We think that radio duty cycle requirement is one of the challenges for multi-hop topologies. Link maintenance will become harder if radio duty cycle (sending periodic beacons or polling nodes) is applied, implying delays in link information propagation in the network. In addition, end nodes near the sink need to transmit for longer time, needing higher duty cycle compared to other nodes that reside at the leaf of network. We think that an average network duty cycle approach, where nodes’ duty cycle will depend on how far from the sink node is, can be a solution. However, the overall average network duty cycle should stay below 1%. In such an approach, nodes near the sink might have duty cycle higher than 1%, while nodes at the boundary of network will have much lower than 1% duty cycle in order to keep the network average under 1%.

### 8.4. Multi-Modal Networks

Performance of heterogeneous IoT applications can be improved by usage of multi-technology networks. Different technologies have their pros and cons in terms of offered throughput, power consumption, covered distances, traffic latencies, etc. Different heterogeneous application can benefit by on-the-fly smart selection of communication technology based on their requirements. To this end, LoRaWAN is seen as one of the possible technology, that, in addition to other technologies such as Wi-Fi, NB-IoT, SigFox or other LPWANs, can improve flexibility and reliability of the communication [24,111,112,127,128,129,130,131].

In [127], a dual-technology (LoRaWAN and NB-IoT) node was prototyped and its communication feasibility over both networks was demonstrated. The NB-IoT network can be used for emergency traffic or to close the loop for auction downlink traffic in different use-cases. In [128], feasibility of using LoRaWAN and NB-IoT for demand-side management of energy system was demonstrated together with a possible network architecture.

In [111,112], a network architecture based on multi-modal nodes has been presented for collecting environment sensor data. For short range communication, IEEE 802.11n is used, while LoRa is used for long distance communication. In [129], a Smart City test-bed has been deployed by using multi-modal gateways that include a number of technologies: IEEE 802.1ac, DASH7, Bluetooth (Low Energy), IEEE 802.15.4, IEEE 802.15.4g and LoRa.

In [130], author presented a framework for LoRa and IEEE 802.11ah inter-working with focus on resource management, network selection and support for mobile nodes, while, in [131], a multi-modal network architecture was presented. In [131], network architecture is composed of a number of multi-modal IoT device (LoRa, SigFox, NB-IoT, and DASH7), several independently operated wireless access networks and the virtual network operator. Virtual network operator offers a unified managing interface for multi-modal network devices and makes possible the device handover from one network to another. Multi-modal end nodes implement the network convergence layer on top of different MAC and PHY layers offering an abstraction toward node’s application. In [58], a demo implementation of such an approach is presented, where a CoAP-UDP-IPV6 stack in combination with a custom SCHC adaptation layer on top of LoRaWAN and Dash7 MAC layer. Although the logic layer for switching the technology is missing, the architecture is promising.

As it is stated in [131] there are several challenges for multi-modal network implementation. Protocol stack design and management is one of the challenges. First steps in SCHC specifications are currently being done; however, techniques for how rules and static context are installed are still missing. In addition, another concern is application security over different technologies. Finally, different technology detection mechanisms should be implemented for different used technologies that makes the solution hard and not scalable. Moreover, different detections mechanisms might have different performance in terms of detection latency. This will make the switching from one to another technology slower compared to the reverse direction.

## 9. Tackling LoRaWAN Challenges and SWOT Analysis

LoRaWAN has its own advantages and disadvantages, as presented with the detailed literature review in the previous sections. LoRaWAN feasibility depends on the application type that is used for. In this section, we try to summarize the open challenges that are not solved with the current LoRaWAN standard. Such challenges can be solved if a number of already published research studies are considered. Some proposals that do not require big changes in standard can be easily included. We will divide the LoRaWAN challenges into three groups that are related with communication scalability and reliability, security issues and malperforming of standard mechanisms.

### 9.1. Communication Scalability and Reliability

Communication scalability in uplink was shown to be a bottleneck for dens LoRaWAN deployments. The number of packet losses increases with the number of end nodes in network or increase of traffic load. In addition, the average power consumption per end node will increase too, decreasing the battery lifetime below the acceptable thresholds. This is due to Aloha-based access scheme that LoRaWAN uses.

Apart from parameter tuning of ADR mechanism in current LoRaWAN, scalability can be increased with transmission synchronization solutions for LoRaWAN. Such solutions include slotted Aloha access scheme for uplink [109], beacon-based time synchronized uplink communication [105], any polling based techniques [112] or even more fine-grained scheduling possibilities [108]. The last method will increase also the reliability, as scheduled traffic is not interfered by other LoRaWAN transmissions. A step further compared to [108] can be to coordinate transmissions from different network technologies to decrease inter-technology interference in LoRaWAN.

Usage of confirmed traffic increases the spectrum occupancy, as many packets are retransmitted due to lack of downlink confirmation. LoRaWAN standard [9,11] specifies that the confirmation has to be transmitted in the receive windows following the uplink transmission. However, the absence of a confirmation does not mean that the uplink packet was lost. Most likely, the absence of confirmation is related to gateway duty cycle. Thus, it is desirable that the confirmation traffic to follow the same mechanism as it is followed in ADR. The end node will wait for the confirmation in the next *D* uplink packets. This will give possibility to gateway to confirm later the uplink traffic, releasing the condition of immediate confirmation. Moreover, the confirmation can be a group confirmation for a group of previous uplink packets.

Radio duty cycle rule is another factor that decrease LoRaWAN scalability (valid only for Europe). Moreover, radio duty cycle is the main factor for low downlink scalability. Implementation of carrier sensing mechanism in gateways will dismiss the need for radio duty cycle in downlink [52,53]. Carrier sensing mechanism in gateways will ease the duty cycle requirements, however, due to large coverage, hidden-node issues cannot be tackled. However, such a mechanism will not ensure high reliability in downlink. Collision avoidance based techniques [111,112] can be used to improve reliability of downlink communication in expense to higher average power usage for end nodes. Another impact in downlink scalability is the packet collision between uplink and downlink traffic. Decoupling the UL/DL communication in different sub-bands will avoid such a collision [110]. Moreover, by using only high-power high duty cycle channel in the first receive window for downlink it will improve downlink scalability and reduce power usage, as shown in Section 9.2.

Another solution that can overcome the duty cycle negative impact in the downlink traffic is to co-locate multiple gateways to increase the duty cycle in downlink. The co-located gateways need to be managed by a central manager that will control the usage of channels as well as SFs in downlink to avoid collisions. In addition, with advancement of electronics, simultaneous transmissions can be possible using different SFs and different channels by single gateway. A time-power multiplexing approach [110] can be used to decrease the gateway duty cycle too.

In some highly dense networks, ADR scheme can end up in a situation where most of the end nodes have switched to the highest SF, increasing the collision possibility at maximum. Thus, in such cases, it is desirable to have a static assignment of SFs. By keeping a share of end nodes that have a static SF and others that will use ADR, the negative impact of ADR in highly congested networks can be decreased.

### 9.2. Power Usage Issues

Low power usage by end nodes is one of the main characteristics that LoRaWAN offers. The power usage is decreased by letting the end node sleep most of the time. However, in congested networks, this is not the case, as end nodes will end up retransmitting many packets or will be locking to uplink traffic in receive windows. However, in the current LoRaWAN standard [11], there are some improvements that can be done to even further decrease the power consumption.

As noticed in [98], much of the node power is used during receive windows when they lock to uplink frames. This can be avoided by using two different SYNC words at the end of programmable part of preamble for uplink and downlink. The end node will stop listening for a packet in any of the receive windows if the SYNC word is of an uplink packet. Thus, end node will be on receiving mode only for the length of programmable part of the preamble that is normally 6–8 symbols long. Another approach is to use totally different preambles for uplink and downlink traffic [98]. This approach will decrease the power usage even further.

Under heavy traffic conditions and networks that are near to congestion, most of the first receive windows are missed by network, mainly due to duty cycle [40]. This is obvious, keeping in mind the low duty cycle (1%) of sub-band channels that can be used for the first receive window. Thus, another unnecessary power usage happens when node opens the first receive window and does not receive any packet. To avoid this, it is beneficial if the first receive window will use the SF of the uplink communication and high-power high duty cycle channel, while the second receive window can be transmitted using the same SF and channel that uplink communication used. Note, that we do not propose to use higher SF in any of the receive windows assuming symmetric links. If the end node sensitivities are more relaxed compared to the gateway, end node need to inform with the uplink packet gateway for unsymmetrical link. In that case, the end node will open only RW2 and the downlink in RW2 uses SF12. Such a scheme will decrease even further the power usage of end devices during receive windows.

### 9.3. Security Challenges

Several security challenges and vulnerabilities have already been addressed by the new standard [11]. The missing of end-to-end message integrity check leaves application payload data integrity check unprotected. This vulnerability is not addressed with the new standard. The malicious node in-between network server and application server can perform bit flipping attacks and these attacks can go undetected. Even worse, this can end in confidentiality compromise of application data if certain changes in application data provoke observable behavior to the attacker. One solution that can be used to ensure end-to-end MIC is to add another MIC between network server and application server. A single key can be used to generate MIC and check it between network and application server independent of the end node traffic.

New LoRaWAN standard specifies the possibility for backward compatibility. This means that, when one of the ends, network server or end device, uses old specification standard, then they both fall back to version v1.0. In such a case, all the vulnerabilities of old standard are possible [132].

### 9.4. SWOT Analysis of LoRaWAN

In this section, we do a SWOT (strengths, weaknesses, opportunities and threats) analysis of LoRaWAN.

LoRaWAN main strengths are that it offers cheap end devices and possibilities of deploying private networks. The latter characteristics remove the need for subscription (such as in SigFox or NB-IoT), decreasing the operational costs for dense IoT application deployments. Other strengths include large coverage with single gateway and low power operation for end nodes. However, such characteristics can be achieved with other network solutions, e.g., SigFox.

The main current weaknesses include security issues (especially for LoRaWAN v1.0), network scalability and malfunction of ADR mechanism in congested traffic. However, there are a number of opportunities to enhance and improve such issues. Many security vulnerabilities are already addressed with LoRaWAN v1.1, except bit flipping attack between NS and AS. Regarding network scalability, different opportunities can be exploited, such as low-power transmission synchronization, low-overhead transmission scheduling, listening-before-talk mechanisms for duty cycle exemption and other more intelligent MAC schemes.

Not suitable for downlink traffic can threaten LoRaWAN’s application in cases when closed-loop communication is needed. This includes many sensors and actuators network when it is crucial to support downlink too. The main threat will continue to be outsider interference as LoRaWAN operates in unlicensed bands. Different techniques to quantify and alleviate it should be considered in the future.

Table 8 lists the SWOT analysis for LoRaWAN.

## 10. Conclusions

In this paper, a literature review for LoRa and LoRaWAN is presented. It includes the most relevant papers published between 2015 and September 2018 regarding LoRa and LoRaWAN technological aspects and improvements. First, a detailed tutorial on how LoRa and LoRaWAN function is presented. Next, the paper offers a structured review by classifying studies that focus on the physical layer performance and network level performance. It also includes the IoT applications and deployments that already use LoRaWAN as the underlying wireless network technology. In addition to this LoRaWAN literature review, other proposed solutions that make a significant extension to the LoRaWAN standard are reviewed. Whenever possible, a comparison between each of the considered solutions is made, including a detailed list of the advantages and disadvantages each solution has. At the end, a set of challenges that still need to be addressed in LoRaWAN is detailed, together with possible approaches on how to tackle those challenges. Finally, the paper summarizes the complete analysis, presenting a SWOT analysis of LoRaWAN.

## Figures and Tables

**Table 1:** LoRaWAN keys.

Key	Description	Required in Joining Type		Generated from or Stored Beforehand
		OTAA	ABP	
LoRaWAN v1.1				
Keys needed before activation				
NwkKey	Is used to calculate MIC for join-request packets, encrypt join-accept packets, and derive all NTW session keys.	Yes	No	Stored beforehand
AppKey	Is used to derive AppSKey	Yes	No	Stored beforehand
JSIntKey	Is used for MIC of rejoin-request and join accept packets	Yes	No	Generated from NwkKey and DevEUI
JSEncKey	Is used to encrypt join-accept triggered by rejoin-request	Yes	No	Generated from NwkKey and DevEUI
Keys needed after activation				
FNwkSIntKey	Is used for calculate MIC of part of it of all uplink data packets	Yes	Yes	Generated from NwkKey and join-accept message
SNwkSIntKey	Is used to verify MIC of all downlink data packets and calculate part of MIC of uplink packets	Yes	Yes	Generated from NwkKey and join-accept message
NwkSEncKey	Is used to encrypt all downlink and uplink MAC packets	Yes	Yes	Generated from NwkKey and join-accept message
AppSKey	Is used to encrypt/decrypt payload of data packets	Yes	Yes	Generated from AppKey and join-accept message
Identifiers				
JoinEUI	64-bit globally unique application ID that identifies the join server	Yes	No	Stored beforehand
DevEUI	64-bit globally unique device ID by the network server	Yes	No	Stored beforehand
DevAddr	32-bit unique device address in the current network	Yes	Yes	Received by join-accept message
LoRaWAN v1.0				
Keys needed before activation				
AppKey	Is used to derive AppSKey and NwkSKey and to calculate MIC for join-request message.	Yes	No	Stored beforehand
Keys needed after activation				
NwkSKey	Is used to encrypt all MAC packets only and to calculate MIC of data packets	Yes	Yes	Generated from AppKey and join-accept message
AppSKey	Is used to encrypt/decrypt payload of data packets	Yes	Yes	Generated from AppKey and join-accept message
Identifiers				
AppEUI	64-bit globally unique application ID	Yes	No	Stored beforehand
DevEUI	64-bit globally unique device ID by the network server	Yes	No	Stored beforehand
DevAddr	32-bit unique device address in the current network	Yes	Yes	Received by join-accept message

**Table 2:** LoRaWAN applications.

Study	Filed of Application	Studied LoRaWAN Performance Indicators		
		Path Loss	PLR	Power Consumption
[12]	Medical	•	•	
[13]	Medical	•	•	•
[14]	Agriculture			
[15]	Agriculture	•		
[16]	Agriculture			•
[17]	Agriculture			•
[18]	Sensor Networks	•	•	
[19]	Sensor Networks	•		
[20]	Traffic Monitoring		•	•
[23]	Localization	•		
[26]	Tele-metering	•	•	
[28]	Smart Grid			
[24]	Smart City	•	•	

**Table 3:** LoRaWAN deployed setups.

Study	Environment	Studied LoRaWAN Performance Indicators				Comments
		Path Loss	PLR	Throughput	Delay	
[32]	Outdoor	Yes	Yes	No	No	@2 km distance using SF12 RSSI > −90 dBm; PLR = 12%
[33]	Outdoor	Yes	No	No	No	@2 km distance coverage only using SF12
[34]	Outdoor	Yes	Yes	Yes	No	@2 km distance using SF12 PLR = 3%
[18]	Outdoor-Indoor	Yes	Yes	No	No	@2 km distance RSSI > −100 dBm AXK rcp = 42%
[35]	Indoor	Yes	Yes	Yes	Yes	@60 m distance with SF12 RSSI > −100 dBm PLR = 2%
[36]	Indoor	Yes	Yes	No	No	@60 m distance with SF12 RSSI > −100 dBm PLR = 2%
[37]	Indoor	Yes	No	No	No	@~32 m distance RSSI > −75 dBm
[39]	Industrial	Yes	Yes	No	No	@~190 m distance RSSI > −100 dBm PLR < 1/%
[24]	City Outdoor	Yes	No	No	No	

**Table 4:** LoRaWAN simulators.

Study	Environment	Included Features					
		Multi GW	Uplink Confirmed	Donlink Traffic	Downlink Confirmed Traffic	MAC Commands	Phy Model
[40]	NS3	Yes	Yes	Yes	Yes	No	All interference based
[42]	NS3	Yes	Yes	Yes	Yes	Yes	Power difference based
[41]	Python	Yes	No	No	No	No	Power difference based
[50]	Python	Yes	No	Yes	No	No	Power difference based
[51]	Python	Yes	No	No	No	No	SIR based with log-normal channel fading
[48]	NS3	No	No	No	No	Yes	Received power based
[53]	NS3	Extend the LoRaWAN module in [48] with p-CSMA based MAC protocol					
[24]	C++	No	Yes	No	No	No	SNR based

**Table 5:** Proposed system models for LoRaWAN.

Study	Model	Considered Interference							
		Dominant Co-SF	Cumulative Co-SF	Dominant Inter-SF	Cumulative Inter-SF	Co and Inter-SF	Path Loss Model	Channel Fading	Other Interference
[34]	Mathema.	No	No	No	No	No	None	No	No
[69]	Mathema.	Yes	No	No	No	No	Yes	No	No
[70]	Mathema.	Yes	No	No	No	No	Yes	No	No
[71]	Mathema.	Yes	No	No	No	No	Yes	No	No
[72]	Mathema.	Yes	Yes	No	No	No	Yes	No	No
[25]	Mathema.	Yes	Yes	No	No	No	Yes	Yes	Yes
[73]	Mathema.	Yes	Yes	Yes	Yes	Yes	Yes	Yes	No
[77]	Mathema.	Models only the network activation procedure							
[75]	Empir.	Yes	No	No	No	No	Yes	No	No
[47]	Empir.	Yes	No	No	No	No	No	No	No
[68]	Empir.	Yes	Yes	Yes	Yes	Yes	Yes	No	No
[40]	Simu.	Yes	Yes	Yes	Yes	Yes	Yes	No	No

**Table 6:** Power consumption modeling for LoRaWAN.

Study	Model	Traffic Type Considered		
		Uplink Only	UL ACKed	Downlink
[79]	Mathematical	Yes	Yes	No
[80]	Empirical	Yes	No	No
[82]	Simulation	Yes	Yes	No
[83]	Empirical	Yes	No	No

**Table 7:** LoRaWAN security aspects research.

Study	Studied Security Aspects	Description
[87]	Data replay attacks for ABP nodes, Eavesdropping, Bit flipping attack.	Re-keying after every reset of counter overflow. Replace the counter with a nonce for AES counter mode. Add a MIC for application layer.
[88]	Key management issues.	Add proxy nodes to drive a reputation system enhancing the LoRaWAN security
[90]	Replay attack and unique DevNonce	Use of sequential DevNonce.
[91]	Replay attack	In addition to DevNonce use RSSI to determine the reply attacks.
[92]	ATAA join-procedure security	Use hybrid crypotosystem including asynchronous cryptosystem. Here the join-request packet is encrypted using AppKey.
[93]	Replay attack	Masking the join-request packet by a unique token derived from the previous NetSKey.
[94]	Replay attack	Two types of join-request packets. The initial ones packets are sent normally. The non-initial packet’s MIC is generated using previous NetSKey.
[95]	Static Context of NetSKey and AppSKey	It involves session key update mechanism into LoRaWAN based on Ephemeral Diffie–Hellman Over COSE (EDHOC) algorithm. It is shown that the message sizze used in EDHOC case is around 40% than in DTLS case.
[96]	Encryption	Secure Low Power Communication method (SeLPC) that reduces the AES encryption cycles in the end node is proposed. The encryption power is minimized up to encryption power up to 26.2%.

**Table 8:** SWOT analyses table.

Strengths	Weaknesses
- Large coverage in outdoor environments - Low power usage of end nodes - Low complexity of end nodes - Cheap end devices - Private network deployment opportunity - Suitable for monitoring applications	- Security issues - Reply and DoS attacks possible - Ntw security and App security terminates at different points in the network - ADR mechanism performs badly under heavy network load (increase power consumption of end-nodes, and collision rate in network) - Low scalability in DL due to duty cycle.
**Opportunities**	**Threats**
- Power usage can decrease further by modifying DL communication scheme - Low-power traffic synchronization possibilities. - Low-power traffic scheduling possibilities - CSMA schemes to avoid duty cycling in DL.	- Scalability issues in UL under heavy network load. - Interference from other technologies. - Not-suitable for two-way communication.
